# Comprehensive analyses reveal the carcinogenic and immunological roles of ANLN in human cancers

**DOI:** 10.1186/s12935-022-02610-1

**Published:** 2022-05-14

**Authors:** Yanlong Shi, Xinyu Ma, Menglu Wang, Sheng Lan, Haokun Jian, Yue Wang, Qian Wei, Fei Zhong

**Affiliations:** 1 Department of General Surgery, Fuyang Hospital Affiliated to Anhui Medical University, Fuyang, Anhui China; 2 Department of Oncology, Fuyang Hospital of Anhui Medical University, Fuyang, Anhui China; 3 The Second Clinical College Clinical Medicine, Guangzhou Medical University, Guangzhou, Guangdong China; 4 School of Basic Medical Sciences, Xinxiang Medical University, Xinxiang, Henan China; 5 Department of Pathology, Anhui Medical University, Hefei, Anhui China; 6 School of Nursing, Anhui Medical University, HeFei, Anhui China

**Keywords:** Anillin, Cancer, Prognosis, Immune infiltration, Biomarker

## Abstract

**Background:**

Anillin (ANLN) is an actin-binding protein that is essential for cell division and contributes to cell growth and migration. Although previous studies have shown that ANLN is related to carcinogenesis, no pan-cancer analyses of ANLN have been reported. Accordingly, in this study, we evaluated the carcinogenic roles of ANLN in various cancer types using online databases.

**Methods:**

We evaluated the potential carcinogenic roles of ANLN using TIMER2 and Gene Expression Omnibus databases with 33 types of cancers. We further investigated the associations of ANLN with patient prognosis, genetic alterations, phosphorylation levels, and immune infiltration in multiple cancers using GEPIA2, cBioPortal, UACLAN, and TIMER2 databases. Additionally, the potential functions of ANLN were explored using Gene Ontology and Kyoto Encyclopedia of Genes and Genomes analyses. Reverse transcription quantitative polymerase chain reaction and immunohistochemistry were used to determine ANLN mRNA and protein expression in colorectal cancer (CRC), gastric cancer (GC), and hepatocellular carcinoma (HCC) cell lines.

**Results:**

ANLN was overexpressed in various tumor tissues compared with corresponding normal tissues, and significant correlations between ANLN expression and patient prognosis, genetic alterations, phosphorylation levels, and immune infiltration were noted. Moreover, enrichment analysis suggested that ANLN functionally affected endocytosis, regulation of actin cytoskeleton, and oxytocin signaling pathways. Importantly, ANLN mRNA and protein expression levels were upregulated in gastrointestinal cancers, including CRC, GC, and HCC.

**Conclusions:**

Our findings suggested that ANLN participated in tumorigenesis and cancer progression and may have applications as a promising biomarker of immune infiltration and prognosis in various cancers.

**Supplementary Information:**

The online version contains supplementary material available at 10.1186/s12935-022-02610-1.

## Background

With rapid increases in global warming and unhealthy lifestyles, cancer has become a major threat to public health worldwide [1]. Oncogenes are genes that promote the neoplastic transformation of cells [2]; accordingly, oncogenes are often highly expressed in various cancers, and cancer can result in the abnormal expression of many oncogenes [3]. Therefore, analysis of oncogene expression may facilitate the identification of cancer and cancer-related mechanisms and to determine patient prognosis. For example, CD96 mediates various immune responses and is associated with immune cell infiltration and prognosis in patients with melanoma and glioma [4]. The identification of oncogenes has been accelerated by developments in sequencing technology; a growing number of genome-wide datasets are available in public platforms, such as The Cancer Genome Atlas (TCGA) and Gene Expression Omnibus (GEO) databases [5, 6].

As a critical factor involved in cell division, anillin (ANLN) is an actin-binding protein that contributes to cell growth and migration [7]. The localization of ANLN varies as the cell cycle progression; ANLN is mainly localized in the nucleus during interphase and in the cell cortex during mitosis [8]. Notably, ANLN is involved in the occurrence and progression of breast cancer [9] and pancreatic cancer [10], and overexpression of ANLN mRNA and protein is associated with poor survival [11]. Moreover, Jia et al. reported that ANLN may be a therapeutic target in patients with hepatocellular carcinoma HCC owing to its effects on carcinogenesis in HCC cell lines [12]. Although the biological functions of ANLN have been extensively studied, few comprehensive analyses have examined the specific roles of ANLN in various cancers.

Accordingly, in this study, we conducted a pan-cancer analysis of ANLN using TCGA and GEO databases. Subsequently, we systematically explored the relationships of ANLN expression with patient prognosis, genetic alterations, phosphorylation, the immune microenvironment, and gene function in order to uncover the molecular mechanisms of ANLN in cancer. Finally, we verified the upregulation of ANLN in gastrointestinal malignancies.

## Materials and methods

### Analysis of gene expression

The Human Protein Atlas (HPA) database, which includes distribution information for 26,000 types of tissues and cells, was applied to investigate ANLN protein expression (https://www.proteinatlas.org/) [13]. The TIMER2 database, a comprehensive analysis network tool was used to explored ANLN expression from TCGA database and the immune microenvironment with the “Gene_DE” module in tumors and corresponding normal tissues [14].

Based on RNA-sequencing expression data, the Gene Expression Profiling Interactive Analysis (GEPIA2) database was applied to investigate tumors without a control group in the TIMER2 database (http://gepia.cancer-pku.cn/#analysis) [15]. Additionally, the “Expression analysis-BoxPlot” module was used to investigate ANLN expression in tumors and normal tissues based on the following criteria: *P* ≤ 0.01, log_2_|fold change| (FC) = 1. We utilized the “Pathological Stage Plot” module to assess correlations between ANLN expression and clinicopathological stage through transforming the log_2_([transcripts per million] + 1). Additionally, CPTAC in the UALCA database was employed to investigate total protein and phosphoprotein expression by searching ANLN in tumor and corresponding normal tissues (http://ualcan.path.uab.edu/analysis-prot.html) [16]. The Oncomine database, a platform based on microarray data, was employed to conduct a meta-analysis of ANLN expression in some types of cancer, using the following parameters: *P* ≤ 0.05, log_2_|FC|= 1.5. The results are presented as medians and *P* values of medians for each type of cancer.

### Analysis of survival prognosis

The association of ANLN expression with overall survival (OS) and disease-free survival (DFS) was determined using the “Survival Map” module of GEPIA2 in different cancers from TCGA datasets. The thresholds for the low- and high-expressoin groups were set to cut-off-low (50%) and cut-off-high (50%) values, respectively, and log-rank tests were used to validate our hypotheses. The “Survival Analysis” module was used to generate survival plots in GEPIA2.

Kaplan–Meier plotter is a tool that uses different GEO databases for the analysis of OS, distant metastasis-free survival (DMFS), progression-free survival (PFS), relapse-free survival (RFS), first progression (FP), disease-specific survival, and post-progression survival (PPS) [17]. Kaplan–Meier survival plots were generated using 95% confidence intervals, “autoselect best cutoff”, and log-rank *P* values.

### Analysis of genetic alteration

Using the cBioPortal website (https://www.cbioportal.org/), we applied the “TCGA Pan Cancer Atlas Studies” module to investigate genetic alterations in *ANLN* [18]. We used the “Mutations” module to explore the mutation site information for the *ANLN* gene. Furthermore, we also obtained data on OS, RFS, PFS, and DFS to assess the effects of *ANLN* genetic alterations using the “comparison” module. Kaplan–Meier plots are presented using log-rank *P* values.

### Analysis of phosphorylation

The ID “ANLN_HUMAN” was entered in the SMART database to obtain ANLN protein domains and phosphorylation sites (http://smart.embl-heidelberg.de/smart/). We then further analyzed phosphorylation sites and ANLN protein expression in different cancers using data from the UACLAN database. The standard deviation between the tumor sample and the median was represented by the z-value.

### Analysis of the tumor immune microenvironment

The “immune gene” module of TIMER2 was used to investigate the association between ANLN expression and immune cells, including CD4^+^ T cells, CD8^+^ T cells, B cells, macrophages, neutrophils, natural killer (NK) cells, and cancer-associated fibroblasts, in different types of tumors. *P* values and partial correlation values were obtained using Spearman rank correlation tests with purity adjustment. Student’s t tests were applied for comparisons between two groups, and analysis of variance was used for comparisons of more than two groups. Pearson’s correlations were used to detect the strength of differences between certain variables.

### Analysis of ANLN-related gene enrichment

The STRING website was used with the following thresholds: protein name, “ANLN”; and organism, “*Homo sapiens*” (https://string-db.org/). The following primary threshold values were set: minimum required interaction score, “low confidence (0.150)”; meaning of the network edge, “evidence”; maximum number of interaction objects to display, “no more than 50 in the first shell”; and source of active interaction, “experiment”. We then downloaded 50 ANLN-binding proteins verified by experiments.

Next, the “Similar Gene Detection” module from the GEPIA2 database was used to obtain the top 100 ANLN-related genes. Pearson correlation analysis was applied to evaluated associations between ANLN and selected genes in the “correlation analysis” module. Furthermore, the ‘Gene_Corr’ module of TIMER2 was used to generate a heatmap for the above genes.

Intersection analysis of ANLN-binding and interacting genes was then performed using the Venn diagram viewer Jvenn [19]. We combined the above two cohorts of data for the Kyoto Encyclopedia of Genes and Genomes (KEGG) pathway analysis with the following parameters in the DAVID database: identifier, “OFFICIAL_GENE_SYMBOL”; and species, “*Homo sapiens*”. Visualization was performed using the R packages “tidyr” and “ggplot2”. Finally, the R package “clusterProfiler” was applied for Gene Ontology (GO) enrichment analysis. Data for molecular functions were visualized as cnetplots, with the following parameters: circular = F, colorEdge = T, node_label = T. In two-tailed tests, *P* values less than 0.05 were considered statistically significant.

### Cell culture

HCT116 and SW480 human colorectal cancer (CRC) cells and NCM460 human normal colonic epithelial cells were purchased from Cell Bank (Shanghai, China) and Procell Life Science (Wuhan, China), respectively. AGS and 7901 human gastric cancer (GC) cells, GES human gastric mucosa epithelial cells, HepG2 and Huh human HCC cells, and LO2 human liver cells were obtained from the Central Laboratory of the First Hospital Affiliated to Anhui Medical University. Cells were cultured in Dulbecco’s modified Eagle’s medium (HyClone) containing 10% fetal bovine serum (VivaCell, Shanghai, China). The cells were incubated at 37 °C in a cell culture incubator with an atmosphere containing 5% CO_2_.

### Reverse transcription quantitative polymerase chain reaction (RT-qPCR)

Total RNA was extracted from cells using TRIzol reagent (Takara, Shiga, Japan). Reverse transcription of cDNA was performed using a Primescript rt kit (Takara) with the following protocol: 37 °C for 15 min, 85 °C for 5 s, and 4 °C for 2 min. *ANLN* expression levels were evaluated by qPCR using SYBR Green qPCR Mix (Takara) with the following protocol: predenaturation at 95 °C for 30 s, 40 cycles of denaturation at 95 °C for 5 s and annealing and extension at 65 °C for 30 s, 95 °C for 10 s, and 65 °C for 5 s. The following primers used were: *GAPDH* forward, 5′-CTCACCGGATGCACCAATGTT-3′ and *GAPDH* reverse, 5′-CGCGTTGCTCACAATGTTCAT-3′; *ANLN* forward, 5′-CAAGATGTATCCAATGACT-3′ and *ANLN* reverse, 5′-TGACTGAAGAATGAATGTT-3′. The relative expression of *ANLN* was determined using the 2^−ΔΔCt^ method.

### Immunohistochemistry of validation

We explored the protein expression of ANLN in gastrointestinal tumors and corresponding normal tissues in “tissue” and “pathology” of modules. All images from immunohistochemical experiments were obtained from the HPA database, and specific patient information was listed. Regents: Antibody HPA005680 (Atlas Antibodies Cat#HPA005680, RRID: AB_2667388) (1:100); Antibody HPA050556 (Atlas Antibodies Cat#HPA050556, RRID: AB_2681175) (1:450); Antibody CAB033902 (Santa Cruz Biotechnology Cat#sc-67327, RRID: AB_2058302) (1:125); Antibody CAB062547 (Atlas Antibodies Cat#AMAb90660, RRID: AB_2665622) (1:100).

### Statistical analysis

Student’s t tests were used to assess *ANLN* gene expression data obtained from the TIMER, GEPIA, and Oncomine databases. The prognostic roles of ANLN were estimated using GEPIA and Kaplan–Meier plotter. Hazard ratios and *P* values or log-rank *P* values were used for comparing OS, RFS, DFS, and DMFS in high- and low-risk groups or altered and unaltered groups. Correlations between ANLN expression and immune infiltration were analyzed using Spearman’s analysis. Differences in ANLN expression between two groups and among multiple groups were analyzed using Student’s t tests and analysis of variance, respectively. Results with *P* values less than 0.05 were considered statistically significant.

## Results

### ANLN expression profiles in normal and tumor tissues

**Fig. 1:**
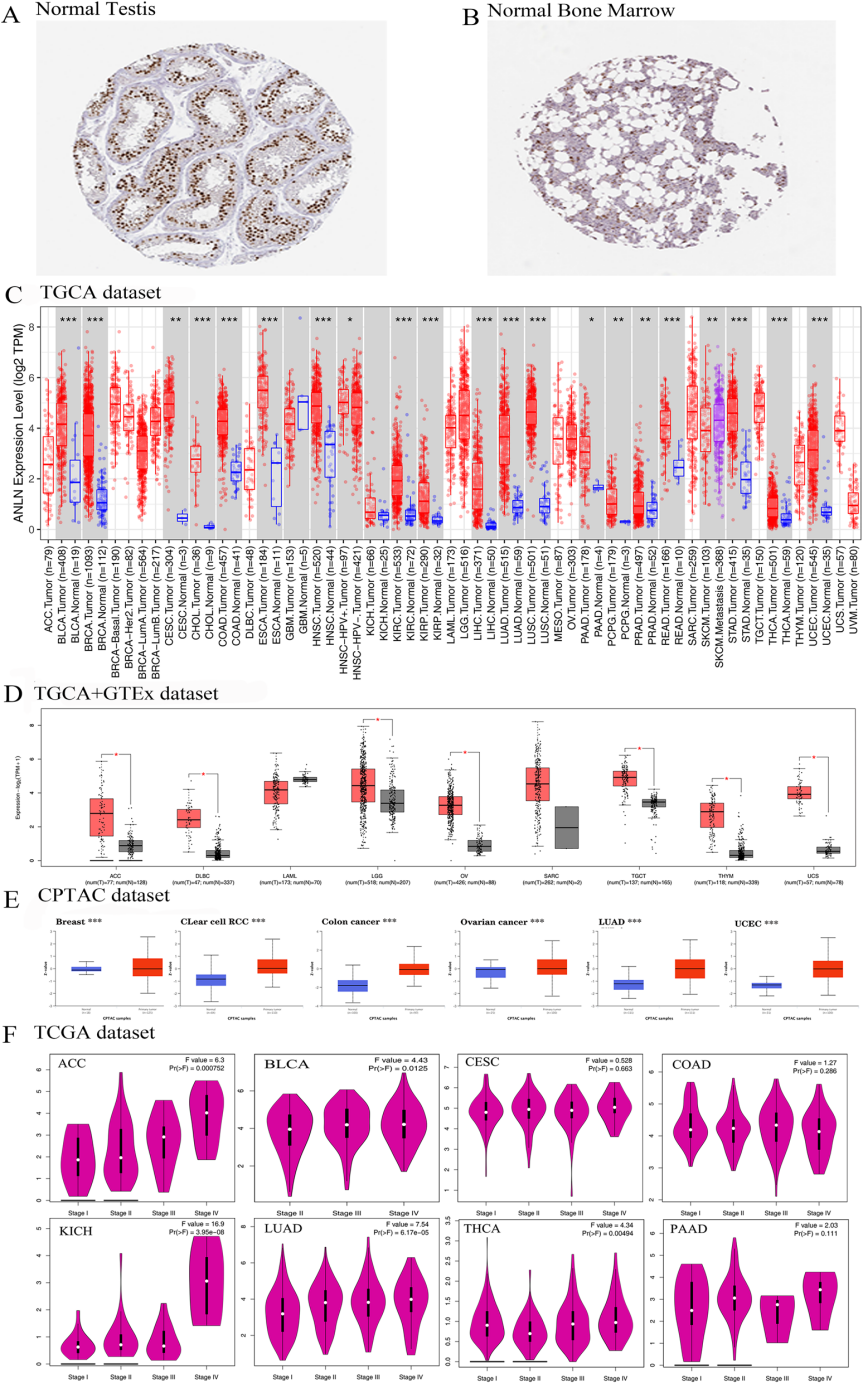
ANLN expression and its correlation with clinicopathological features. **A** The expression of ANLN mRNA in human normal testis tissues. **B** The expression of ANLN mRNA in human normal bone marrow tissues. **C** Analysis ANLN expression in various tumors or certain tumor subtypes in TIMER2 database. **D** Analysis of ACC, DLBC, LAML, LGG, OV, SARC, TGCT, and UCS expression between TCGA tumor tissues and GTEx database the corresponding normal tissues. **E** Analysis of ANLN total protein expression between breast cancer, clear cell RCC, colon cancer, ovarian cancer, LUAD, UCEC and corresponding normal tissues in CPTAC dataset. **F** Analysis of ANLN in pathological stages of ACC, BLCA, CESC, COAD, KICH, LUAD, PAAD, THCA. *P < 0.05; **P < 0.01; ***P < 0.001; Log scale adopts Log2 (TPM + 1)

As shown in Fig. 1A and B, ANLN protein was highly expressed in testis and bone marrow tissues, and immunohistochemical analyses obtained from the HPA database suggested that ANLN protein was primarily localized in the cell nucleus. Further analysis of ANLN expression in the TIMER2 database (Fig. 1C) showed that ANLN was upregulated in breast carcinoma (BLCA), breast invasive carcinoma (BRCA), colon adenocarcinoma (COAD), cholangiocarcinoma, head and neck squamous cell carcinoma (HNSC), kidney renal papillary cell carcinoma (KIRP), esophageal carcinoma (ESCA), lung squamous cell carcinoma, kidney renal clear cell carcinoma (KIRC), liver HCC (LIHC), rectum adenocarcinoma, lung adenocarcinoma (LUAD), thyroid carcinoma (THCA), stomach adenocarcinoma (STAD), uterine corpus endometrial carcinoma (UCEC; *P* < 0.001), pancreatic adenocarcinoma (PRAD), cervical squamous cell carcinoma and endocervical adenocarcinoma, skin cutaneous melanoma (SKCM), pheochromocytoma and paraganglioma (*P* < 0.01), and pancreatic adenocarcinoma (PAAD; *P* < 0.05).

Because of a lack of some types of normal tissues in TIMER2 database, we further evaluated ANLN expression in the GTEx database using GEPIA2. The results suggested that ANLN expression in cancer tissues exceeded that in corresponding normal tissues for lymphoid neoplasm diffuse large B-cell lymphoma, brain lower grade glioma (LGG), thymoma (THYM), skin cutaneous melanoma (SKCM), and testicular germ cell tumors (TGCTs). There were no significant differences between tumor and normal tissues for acute myeloid leukemia, kidney chromophobe (KICH), sarcoma, or glioblastoma multiforme (GBM; *P* > 0.05; Fig. 1D).

Total ANLN protein was evaluated using CPTAC datasets; the findings suggested that ANLN was upregulated in BLCA, ovarian cancer, COAD, LUAD, KIRC, and UCEC (*P* < 0.05; Fig. 1E). Furthermore, meta-analysis results confirmed that ANLN was overexpressed in cervical cancer, HNSC, CRC, BLCA, and STAD in the Oncomine database (*P* < 0.05; Additional file 1: Figure S1A–E).

To further investigate ANLN expression, we evaluated the association between ANLN expression and pathological tumor stage using the “Stage Plot” module in GEPIA2. The results showed that ANLN expression was associated with pathological tumor stage in ACC, BLCA, BRCA, KICH, KIRP, LUAD, KIRC, LIHC, and UCS (*P* < 0.05), but not in other tumor types (Fig. 1F; Additional file 2: Figure S2A–D).

### Analysis of the prognostic value of ANLN

**Fig. 2:**
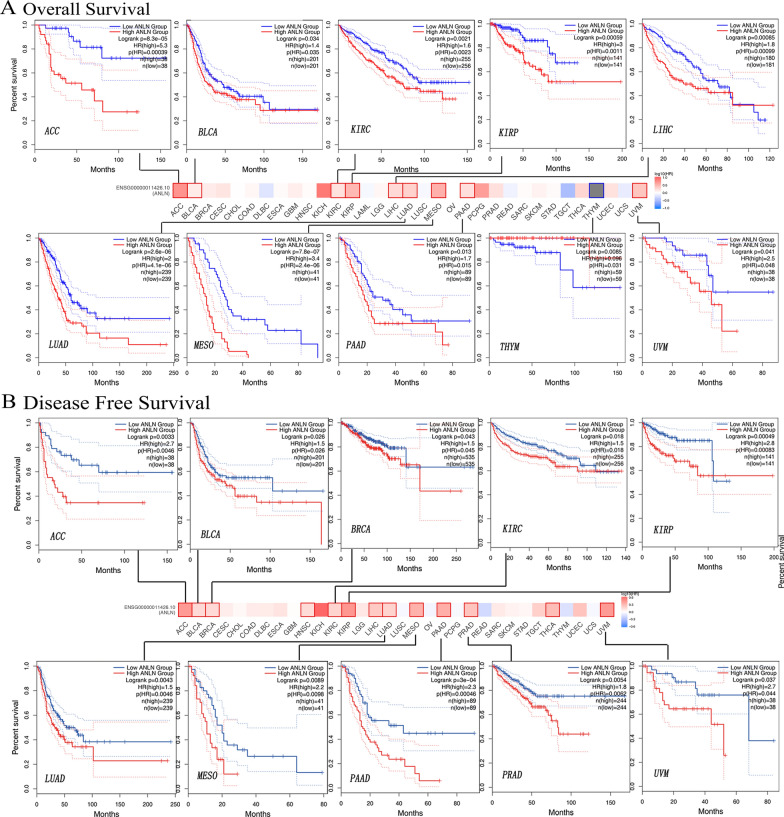
The correlation analysis between ANLN expression and prognosis of cancers in TCGA database using GEPIA2. **A** Overall survival. **B** Disease-free survival. The survival map with positive results were presented

**Fig. 3:**
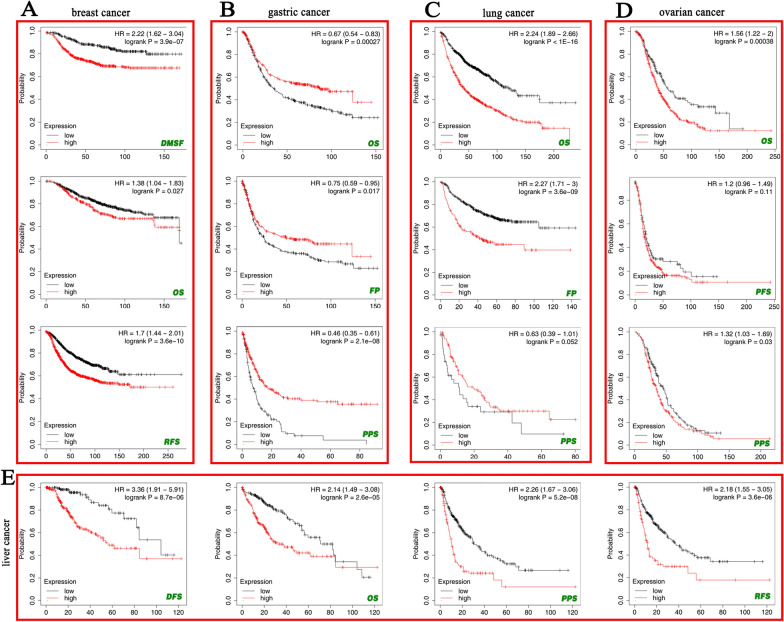
The correlation between ANLN expression and prognosis of certain cancers using the Kaplan–Meier plotter containing OS, RFS, PFS, PPS, DMFS, FP, and DFS. **A** Breast cancer. **B** Gastric cancer. **C** Lung cancer. **D** Ovarian cancer. **E** Liver cancer

Based on our findings regarding the expression of ANLN in different cancer types, patients were segmented into high and low expression groups to investigate the correlation of ANLN expression with prognosis. As shown in Fig. 3A and B, patients with low expression had longer OS and DFS than those with high expression in ACC (*P* = 8.3e-05; *P* = 0.0033), BLCA (*P* = 0.034; *P* = 0.026), KIRC (*P* = 0.0021; *P* = 0.018), KIRP (*P* = 0.00059; *P* = 0.00049), LIHC (*P* = 0.00085; *P* = 0.00046), LUAD (*P* = 2.6e-06; *P* = 0.0043), MESO (*P* = 7.8e-07; *P* = 0.0089), PAAD (*P* = 0.013; *P* = 3e-04), and UVM (*P* = 0.041; *P* = 0.037), suggesting that low ANLN expression was associated with better prognosis. By contrast, in THYM, ANLN upregulation was correlated with longer OS (*P* = 0.0085), but not with DFS (*P* = 0.47; Fig. 2A and B; Additional file 3: Figure S3A and B).

Notably, in patients with breast cancer, low ANLN expression was correlated with better DMFS (*P* = 3.9e-07), RFS (*P* = 3.6e-10), and OS (*P* = 0.027), as demonstrated using Kaplan–Meier plotter (Fig. 3A). By contrast, high ANLN expression was related to better OS (*P* = 0.00027), FP (*P* = 0.017), and PPS (*P* = 2.1e−08) in patients with GC (Fig. 3B). The opposite results were observed for OS (*P* < 0.0001) and FP (*P* = 3.6e−09) in patients with lung cancer (Fig. 3C). In ovarian cancer, high expression ANLN was associated with poor OS (*P* = 0.00038) and PPF (*P* = 0.01; Fig. 3D). Moreover, ANLN expression level was significantly correlated with OS (*P* = 2.6e−05), DFS (*P* = 8.7e−06), RFS (*P* = 3.6e−06), and PPS (*P* = 5.2e−08) in patients with liver cancer (Fig. 3E). Overall, these findings showed that ANLN expression was associated with prognosis in various types of tumors and that high ANLN expression was associated with poor prognosis in most cancers.

### Genetic alterations in ANLN

**Fig. 4:**
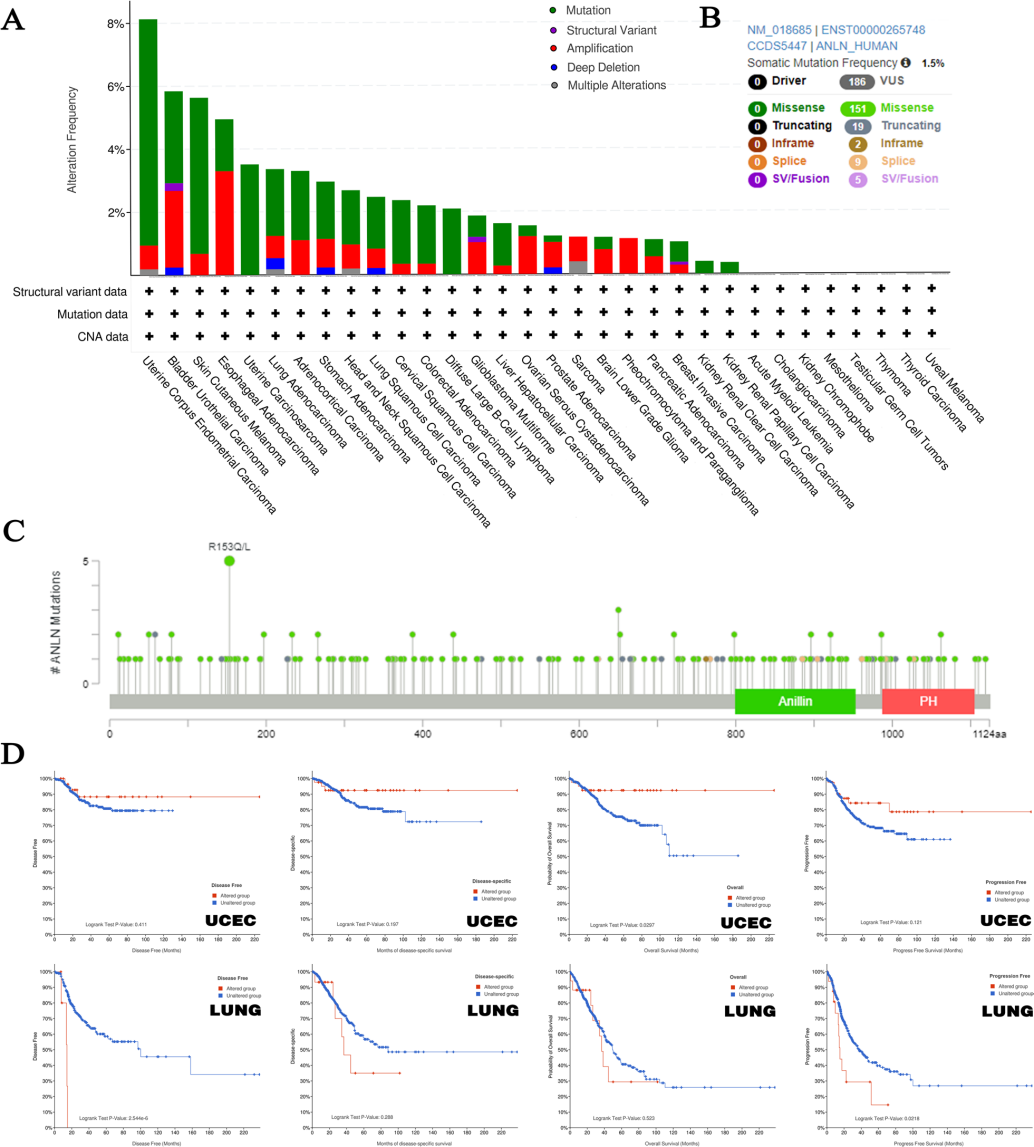
Analysis of ANLN genetic alteration of TCGA cancers the cBioPortal database. **A** The alteration frequency with mutation type. **B** The alteration frequency with mutation site. **C** The correlation between alteration status and prognosis in UCEC and LUNG

Next, the cBioPortal database was used to investigate genetic alterations in ANLN in various types of tumors. We found that the frequency of ANLN alterations was the most common in UCEC (mutated in 7.18% of cases), followed by skin cutaneous melanoma (4.95%). In esophageal adenocarcinoma, 3.3% of cases showed amplification, and amplification was the only alteration type observed in all cases of pheochromocytoma and paraganglioma (Fig. 4A). Moreover, in bladder urothelial carcinoma and LUAD, alterations observed in TCGA datasets included mutations, amplifications, multiple alterations, deep deletions, and structural variants; missense mutations were the main types of ANLN mutations (Fig. 4B). Among 181 mutants, R153Q/L mutation was detected in five cases (uterine serous carcinoma, LUAD, cutaneous melanoma, mucinous adenocarcinoma of the colon and rectum, and HNSC) and induced a frame-shift mutation in ANLN, resulting in truncation of the protein (Fig. 4C). No three-dimensional structure in the ANLN protein was detected at the mutation site (amino acid 153). We then analyzed the association between ANLN genetic alterations and patient prognosis. In patients with UCEC, patients with alterations in ANLN showed improved OS (*P* = 0.0297), but not DSF (*P* = 0.197), DFS (*P* = 0.411), or PFS (*P* = 0.121) in comparison with patients harboring wild-type ANLN (Fig. 4C). By contrast, in patients with LUAD, those without ANLN modifications had longer PFS (*P* = 0.0218) and DFS (*P* = 2.544e-6) than those with ANLN alterations, although no changes in OS (*P* = 0.523) or DSF (*P* = 0.288) were observed.

### Phosphorylation of ANLN

**Fig. 5:**
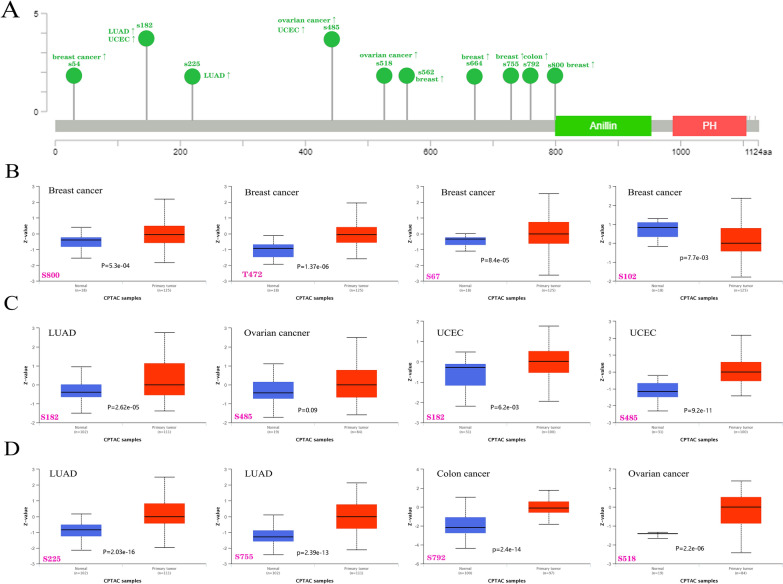
Analysis of ANLN protein phosphorylation in cancers using CPTAC of UACLAN database. **A** Phosphorylation sites with significant differences are marked in ANLN protein (NP_061155.2, S6, S102, S182, S225, S272, S485, S518, S562, S664, S755, S792, S800 sites). **B** Expression of different ANLN protein phosphorylation sites in breast cancer. **C** Expression of ANLN protein phosphorylation sites S182 and S485 in LUAD, ovarian cancer and UCEC. **D** Expression of ANLN phosphorylation sites S225, S755, S792 and S518 in cancers

We then identified significantly different phosphorylation sites in ANLN protein (Fig. 5A) and assessed differences in the phosphorylation levels of ANLN between normal and tumor tissues using the CPTAC database for patients with BLCA, CRC, LUAD, UCEC, and ovarian cancer. Many phosphorylation sites were found to contribute to tumor development and progression. The phosphorylation of ANLN protein was increased at phosphorylation site S182 in LUAD and UCEC and at S485 in ovarian cancer and UCEC (Fig. 5B). Furthermore, many phosphorylation sites showed enhanced phosphorylation in BLCA, including S67, T272, and S800, whereas phosphorylation at S102 decreased (Fig. 5C). In LUAD, CRC, and ovarian cancer, ANLN showed increased phosphorylation at S225, S755, S792, and S518 (Fig. 5D). Furthermore, data from the PhosphoNET database indicated that ANLN phosphorylation at S102, S182, S485, and S518 has been experimentally confirmed [20–23] (Table 1). Further molecular analyses are required to assess the specific mechanisms through which phosphorylation contributes to tumorigenesis.

**Table 1:** Analysis of CPTAC-identified phosphorylation sites of *ANLN* via the PhosphoNET database

Site	Sequence	Experimentally confirmed^#^	Hydrophobicity	P-site similarity score	Maximum kinase specificity	Sum kinase specificity score	Conservation score
S182	PSEEKAASPPRPLLS	17,446,864	− 1.027	− 50.1	599	22,652	17.9
S800	PQSEFMPSKGSVTLS	NA	− 0.593	− 56.7	353	14,866	23.3
S102	SCSPSPVSPQVQPQA	19,276,368	− 0.707	− 55.7	573	23,381	13.3
S67	SCTKPSPSKKRCSDN	NA	− 1.900	− 59.5	328	13,839	23.0
S485	QGVSJTQSLPVTEKV	15,302,935	− 0.567	− 56.1	375	15,798	10.0
T472	QETHCQSTPLKKHQG	NA	− 1.793	− 60.1	270	9245	14.6
S518	ECEMTKSSPLKITLF	18,669,648	− 0.113	59.9	487	20,153	18.8

^*#*^The PMID (PubMed Unique Identifier) information of the publication was provided; NA, not available

### Relationship between immune infiltration and ANLN

**Fig. 6:**
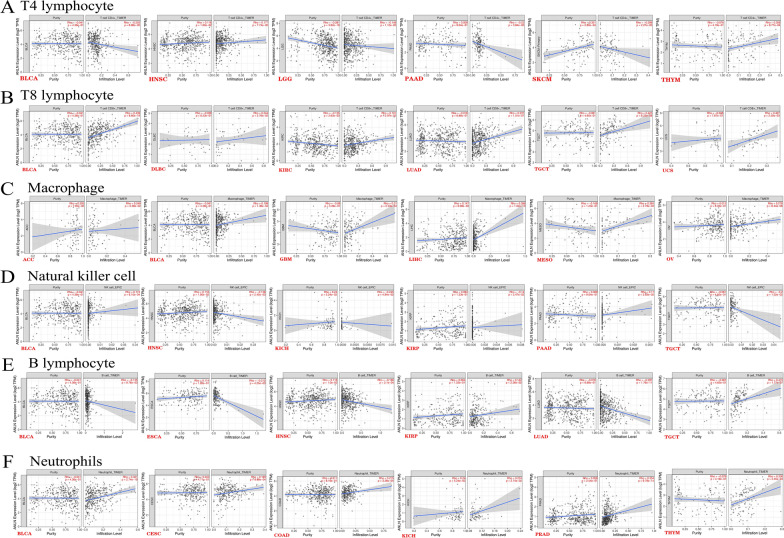
Analysis of the correlation between ANLN expression and immune cells. **A** CD4^+^T cells. **B** CD8^+^T cells. **C** Macrophage. **D** NK cells. **E** B cells. **F** Neutrophils

The tumor microenvironment consists mainly of a mixture of tumor cells and stromal components and is strongly associated with tumorigenesis, invasion, and metastasis [24, 25]. Hence, we next examined whether ANLN expression was related to the tumor microenvironment in various types of cancer by assessing changes in CD8^+^ T cells, CD4^+^ T cells, B cells, NK cells, neutrophils, and macrophage in samples showing variations in ANLN expression in the TIMER, MCPCOUNTER, and TIDE databases. Overall, the expression of ANLN was found to be associated with the infiltration levels of CD8^+^ T cells in 13 tumor types, CD4^+^ T cells in 20 tumor types, B cells in 20 tumor types, macrophages in 20 tumor types, neutrophils in 26 tumor types, and NK cells in 15 tumor types (Fig. 6A–F, Additional file 4: Figure S4, Additional file 5: Figure S5, Additional file 6: Figure S6). Furthermore, ANLN expression was positively associated with immune infiltration of CD8^+^ T cells, neutrophils, and macrophages, but negatively associated with CD4^+^ T cells in BCLA, LGG, PAAD, and SKCM. NK cells were negatively correlated with ANLN expression in HNSC, KICH, and TGCT, and B cells were negatively correlated with ANLN expression in ESCA, HNSC, and LUAD. The results also suggested that ANLN expression was highly correlated with immune infiltration in LIHC; indeed, in LIHC, ANLN expression was positively correlated with tumor purity, CD8^+^ T cells, CD4^+^ T cells, B cells, neutrophils, and macrophages and negatively correlated with NK cells (*P* < 0.05).

**Fig. 7:**
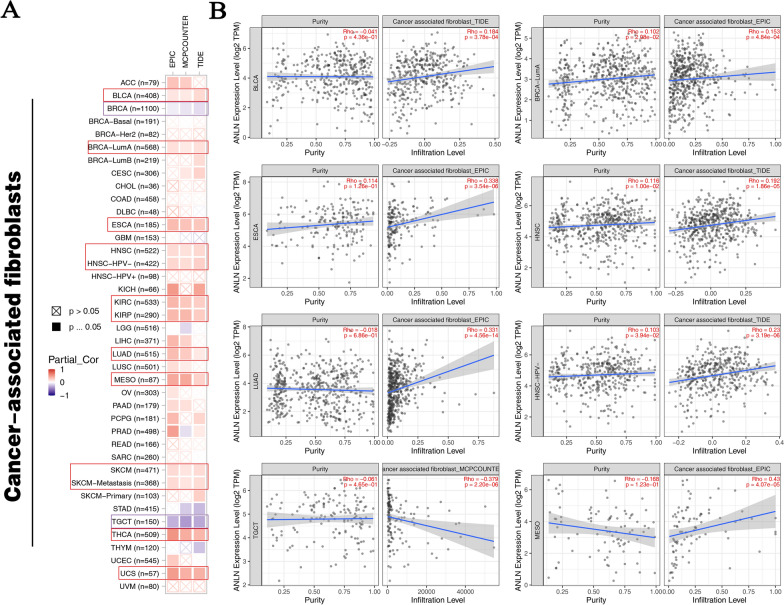
Analysis of the correlation between ANLN expression and immune cell infiltration of cancer-associated fibroblasts in cancers. **A** The heatmap of all tumors in TCGA generated by EPIC, MCPCUNTER, and TIDE algorithm. **B** The scatter plots of cancers generated by one algorithm, including BLCA, BLCA-LumA, ESCA, HNSC, LUAD, HNSC-HPV-, TGCT, and MESO

Cancer-associated fibroblasts are pivotal components of the tumor stroma and participate in modulating tumor-infiltrating immune cells [26]. Importantly, we demonstrated that ANLN expression was positively correlated with cancer-associated fibroblasts in BLCA, BRCA-LumA, ESCA, HNSC, human papillomavirus-negative HNSC, KIRC, KIRP, LUAD, MESO, SKCM, metastatic SKCM, THCA, and USC, but negatively correlated with BRCA and TCGT (Fig. 7A and B).

### Enrichment of ANLN-related genes

**Fig. 8:**
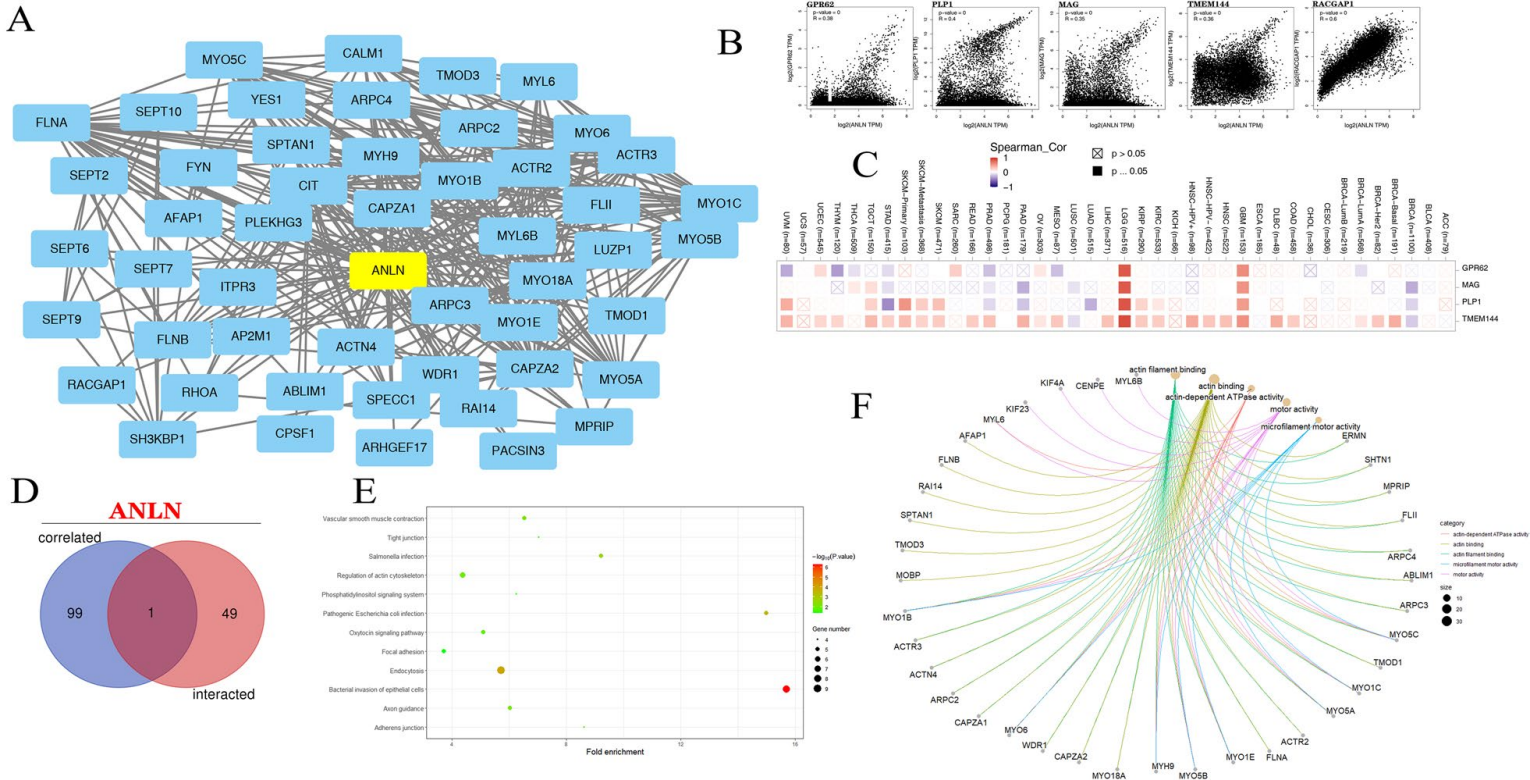
Enrichment analysis ANLN-related genes. **A** The interaction network of 50 ANLN-binding proteins with available experimentally evidenced in the STING. **B** The correlation between ANLN and selected genes, including GPR62, PLP1, MAG, TMEM144, RAGGCP1 using GEPIA2. **C** The corresponding heatmap in the all cancers are presented. **D** Venn diagram of the ANLN-binding and related genes. **E** Analysis of KEGG pathway. **F** The cnetplot for the molecular function based on GO analysis

We screened out ANLN-binding proteins and ANLN co-expression genes for pathway enrichment analyses to elucidate the molecular mechanisms through which ANLN may contribute to cancer occurrence and progression. As shown in Fig. 8A, we acquired 50 experimentally verified ANLN-binding proteins and their interaction networks using the STRING tool. Then, based on tumor expression data from TCGA in GEPIA2, we performed correlation analysis to investigate the top 100 ANLN co-expression genes. As shown in Fig. 8B, ANLN expression was positively correlated with G protein-coupled receptor 62 (GPR62; *R* = 0.38), myelin-associated glycoprotein (MAG; *R* = 0.35), proteolipid protein 1 (PLP1; *R* = 0.4), Rac GTPase activating protein 1 (RACGAP1; *R* = 0.6), and transmembrane protein 144 (TMEM144; *R* = 0.36; all *P* < 0.001). Moreover, heatmap analysis also showed that these genes were positively correlated with ANLN expression in various cancers, including GBM and LGG (Fig. 8C). Further analyses showed that RACGAP1 was identified as both an ANLN-binding protein and ANLN co-expression gene (Fig. 8D).

We then conducted KEGG and GO enrichment analyses for ANLN. KEGG enrichment analyses indicated that the role of ANLN in tumorigenesis was associated with genes involved in endocytosis, bacterial invasion of epithelial cells, regulation of the actin cytoskeleton, and the oxytocin signaling pathway (Fig. 8E). Notably, however, most genes associated with the role of ANLN in tumor progression were related to cellular biology or the microstructure of actin, such as actin binding, actin filament binding, motor activity, ATPase activity, and structural constituents of the cytoskeleton (Fig. 8F).

### Verification the expression of ANLN in gastrointestinal cancers

**Fig. 9:**
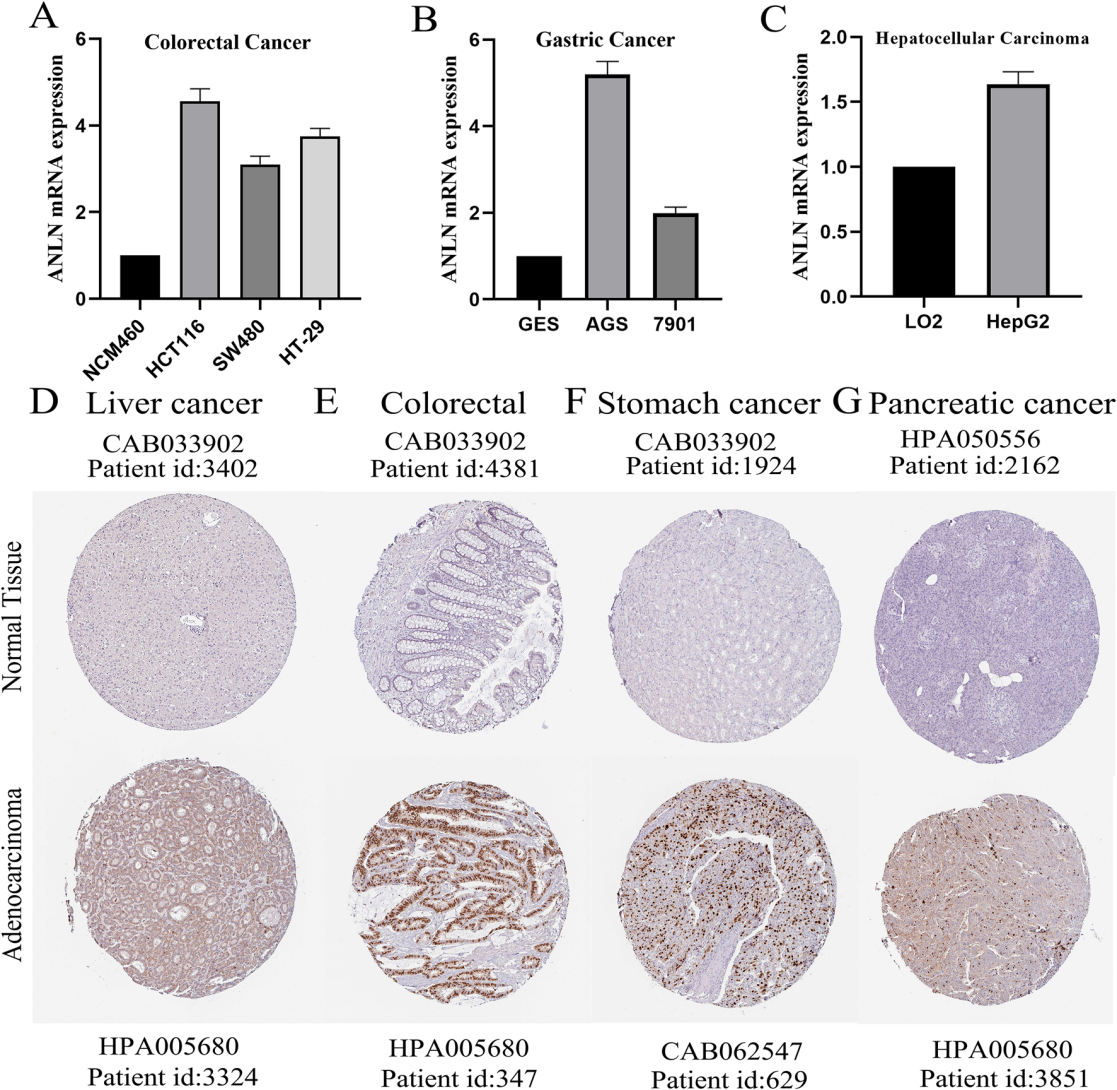
Validation of ANLN mRNA and protein expression in gastrointestinal malignancies. **A–C** qRT-PCR analysis detected the ANLN mRNA expression relative to GAPDH in colorectal cancer, gastric cancer, and hepatocellular carcinoma cell lines. **D–G** The Human Protein Atlas database validated the ANLN protein expression in liver cancer, colorectal cancer, stomach cancer, pancreatic cancer and corresponding normal tissues. *P < 0.05

Next, we assessed the expression of *ANLN* mRNA in HCC, GC, and CRC cell lines using RT-qPCR. The results suggested that *ANLN* mRNA expression was higher in HepG2 HCC cells, 7901 and AGS GC cells, and HCT116 and SW480 CRC cells than in LO2 normal hepatic epithelial cells, GES gastric epithelial cells, and NCM460 colon epithelial cells, respectively (Fig. 9A–C). Additionally, we further confirmed the protein expression of ANLN in gastrointestinal tumors using immunohistochemistry data from the HPA database. In comparison with corresponding normal tissues, *ANLN* was upregulated in HCC, GC, CRC, and pancreatic cancer tissues (Fig. 9D–G). The expression of ANLN protein was predominately localized in the nucleus in GC and CRC and in the cytoplasm and membrane in HCC and pancreatic cancer.

## Discussion

Cancer is a major threat to human health, and studies are urgently needed to identify potential prognostic biomarkers and explore the mechanisms of cancer occurrence and progression. Although ANLN is highly expressed in normal testis and bone marrow, abnormal expression of ANLN has not been found in diseases related to these organs. Many studies have shown that ANLN protein affects various cellular processes, such as cytokinesis, cell cycle, podocyte cell adhesion, and motility [7, 27, 28]. Moreover, ANLN has been shown to have roles in carcinogenesis. For example, ANLN silencing using lentivirus transfection inhibits proliferation, migration, and cell cycle progression in breast cancer cells [29]. However, no pan-cancer analyses of ANLN in various types of tumors has been reported, and it is unclear whether ANLN has critical roles in multiple types of cancer through a common molecular mechanism.

In this study, we found that ANLN was overexpressed in most types of tumors compared with corresponding normal tissues. Furthermore, ANLN expression was found to be associated with pathological stage in ACC, BLCA, BRCA, KICH, KIRC, KIRP, LIHC, LUAD, and UCS, and survival analysis indicated that ANLN upregulation was correlated with poor prognosis in ACC and LIHC. ANLN overexpression in THYM was associated with better OS, but poorer RFS. Overall, ANLN expression was associated with poor prognosis in most types of cancer. However, high ANLN expression was associated with better prognosis in GC; this result may be related to the unique pathological features of GC. Indeed, in a previous study, ANLN was shown to be overexpressed in proliferative gastric tumors compared with aggressive and metabolic gastric tumors [30].

Genetic mutations play essential roles in cancer metastasis and recurrence [31, 32]. In breast cancer, different genetic mutations are associated with specific metastasis sites, and these mutations may therefore represent biomarkers or therapeutic targets in patients with metastatic breast cancer [33]. In this study, our results showed that alterations in *ANLN* were most common in uterine cancer, followed by bladder cancer, UCEC, and SKCM. Moreover, *ANLN* alterations could be protective in UCEC, whereas *ANLN* mutations were associated with shorter RFS and PFS, but not OS, in patients with LUAD. Therefore, as an oncogene, *ANLN* may be a prognostic factor in multiple types of tumors.

The tumor immune microenvironment extensively influences the migration, invasion, and metastasis of various types of cancer cells [34, 35], and immunotherapy has recently been shown to have important roles in the management of patients with cancer by inhibiting the tumor immune microenvironment and thereby exerting antitumor immune activity [36, 37]. In our study, ANLN expression was correlated with CD8^+^ T cells, neutrophils, and macrophages in BLCA, LIHC, KIRP, PRAD, and HNSC. As a vital component of stromal cells, cancer-associated fibroblasts are associated with disease recurrence and chemotherapy resistance in several types of cancer [38]. We found that ANLN expression was associated with cancer-associated fibroblasts in most tumor types. Combined with the results of survival analyses, our findings confirmed that cancer-associated fibroblasts were associated with a poor prognosis in many cancer types, including LIHC and KICH. A previous study also revealed that stromal gene expression was related to a poor prognosis in patients with CRC [39]. Overall, our results indicated that aberrant ANLN expression could alter tumor immunity. Further studies are needed to fully elucidate the molecular mechanisms through which ANLN exerts these effects.

We also evaluated ANLN-binding proteins and co-expressed genes. Our results showed that ANLN-related genes were primarily associated with cytokinesis, cell movement, and cell signaling, consistent with previous studies. Furthermore, we found that ANLN interacted with GPR62, MAG, PLP1, RACGAP1, and TMEM144; RACGAP1 expression was particularly associated with ANLN expression. These glycoprotein-related genes are mainly involved in the regulation of cellular physiological processes [40–43]. Furthermore, RACGAP1 plays key roles in several cellular processes, including differentiation and migration, and its expression is strongly correlated with advanced-stage tumors [44–46]. These results from enrichment analysis established a basis for further exploration of the functions and regulatory mechanisms of ANLN.

There were several limitations to this study. First, the limited number of samples from individual tumors may have led to inaccurate results. Second, we only verified ANLN expression in gastrointestinal cancers, and the functions of ANLN in vivo and in vitro still need to be clarified. Third, more work is needed to evaluate the effects of ANLN on promoting tumor occurrence and progression.

## Conclusion

In conclusion, our results showed that ANLN expression was increased in various types of tumors and that ANLN expression was correlated with prognosis, suggesting that ANLN may be a prognostic indicator for certain cancers, particularly LIHC. Moreover, we further identified the potential molecular mechanisms through which ANLN may modulate immune infiltration, cell division, and cell the cycle. Further studies are needed to validate the potential applications of ANLN in the diagnosis and treatment of cancers.

## Supplementary Information

**Additional file 1: Fig. S1.** Meta analysis on the ANLN expression difference between normal tumor and tumor using the Oncomine. **A** Cervical cancer. **B** Head and neck cancer. **C** Colorectal cancer. **D** Breast cancer. **E** Gastric cancer.

**Additional file 2: Fig. S2.** Expression of ANLN in different pathological stages across cancers. **A** CESC, DLBC, COAD, CHOL. **B** ESCA, HNSC, LUSC, OV. **C** PAAD, READ, THCA, UCEC. **D** STAD, SKCM, UCS, TGCT.

**Additional file 3: Fig. S3.** The correlation analysis between ANLN expression and prognosis of cancers in TCGA database using GEPIA2. **A** Overall survival. **B** Disease-free survival.

**Additional file 4: Fig. S4.** Analysis of the correlation between ANLN expression and immune cells. **A** CD4^+^T cells. **B** CD8^+^T cells.

**Additional file 5: Fig. S5.** Analysis of the correlation between ANLN expression and immune cells. **A** Macrophage. **B** NK cells.

**Additional file 6: Fig. S6.** Analysis of the correlation between ANLN expression and immune cells. **A** B cells. **B** Neutrophils.

## Data Availability

The datasets obtained and explored in this study are available in the GEPIA2, UACLAN, HPA and TIMER2 databases.

## Abbreviations

TCGA: The Cancer Genome Atlas
GEO: Gene Expression Omnibus
ANLN: Anillin
HPA: Human Protein Atlas
GEPIA2: Gene Expression Profiling Interactive Analysis
FC: Fold change
OS: Overall survival
DFS: Disease-free survival
DMFS: Distant metastasis-free survival
PFS: Progression-free survival
RFS: Relapse-free survival
FP: First progression
PPS: Post-progression survival
KEGG: Kyoto Encyclopedia of Genes and Genomes
GO: Gene Ontology
BLCA: Breast carcinoma
BRCA: Breast invasive carcinoma
COAD: Colon adenocarcinoma
HNSC: Head and neck squamous cell carcinoma
KIRP: Kidney renal papillary cell carcinoma
ESCA: Esophageal carcinoma
KIRC: Kidney renal clear cell carcinoma
LIHC: Liver hepatocellular carcinoma
LUAD: Lung adenocarcinoma
THCA: Thyroid carcinoma
STAD: Stomach adenocarcinoma
UCEC: Uterine corpus endometrial carcinoma
PRAD: Pancreatic adenocarcinoma
SKCM: Skin cutaneous melanoma
LGG: Lower grade glioma
THYM: Thymoma
TGCT: Testicular germ cell tumor
KICH: Kidney chromophobe
GBM: Glioblastoma multiforme
GPR62: G protein-coupled receptor 62
MAG: Myelin-associated glycoprotein
PLP1: Proteolipid protein 1
RACGAP1: Rac GTPase activating protein 1
TMEM144: Transmembrane protein 144
