# Supplementary figures and images for: Comprehensive analyses reveal the carcinogenic and immunological roles of ANLN in human cancers
Source: Cancer Cell Int. 2022 May 14;22:188. doi: 10.1186/s12935-022-02610-1 (PMC9107662; doi:10.1186/s12935-022-02610-1)

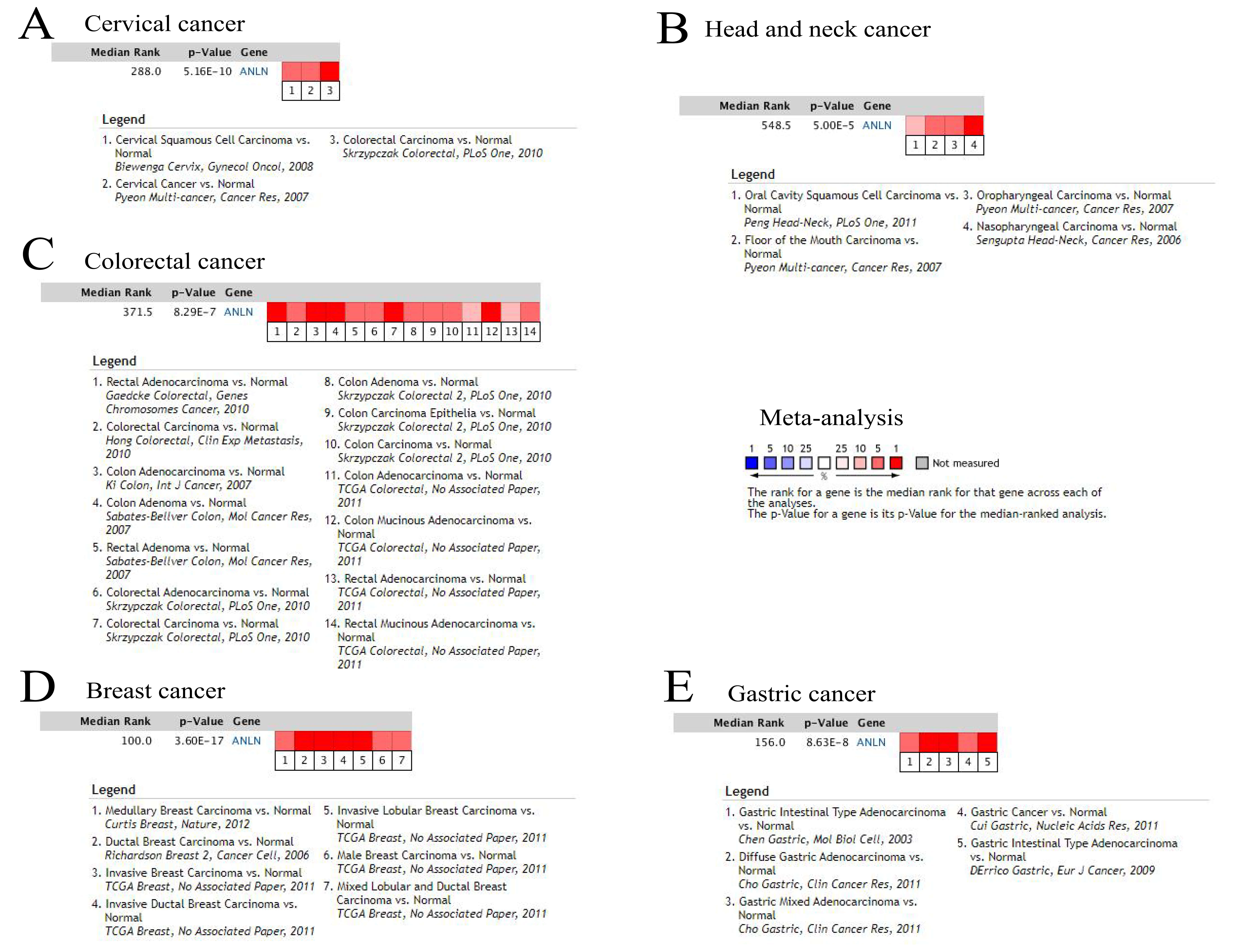

Supplement: Supplementary file 1 — Additional file 1: Fig. S1. Meta analysis on the ANLN expression difference between normal tumor and tumor using the Oncomine. A Cervical cancer. B Head and neck cancer. C Colorectal cancer. D Breast cancer. E Gastric cancer. [file 12935_2022_2610_MOESM1_ESM.tif]

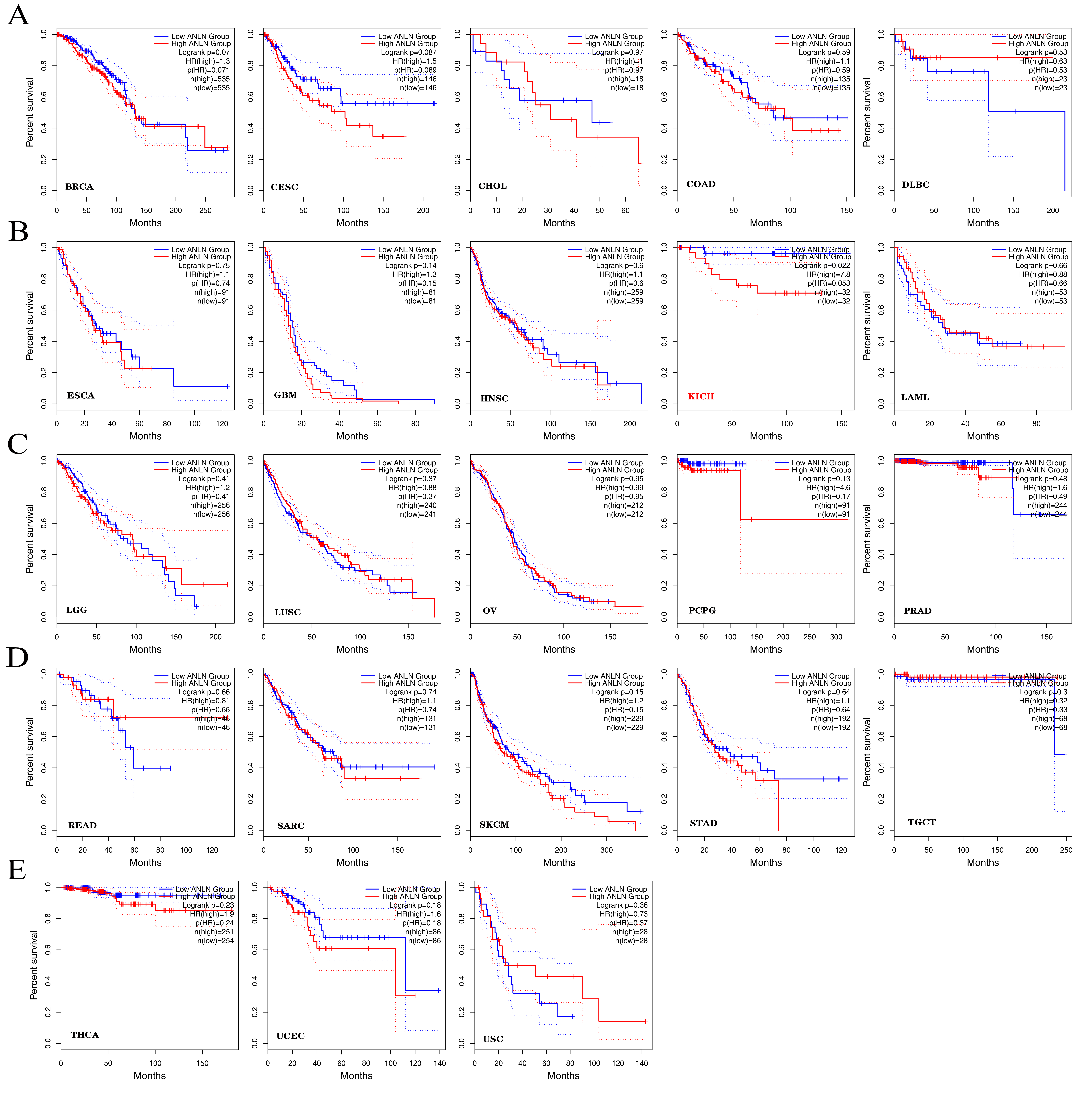

Supplement: Supplementary file 3 — Additional file 3: Fig. S3. The correlation analysis between ANLN expression and prognosis of cancers in TCGA database using GEPIA2. A Overall survival. B Disease-free survival. [file 12935_2022_2610_MOESM3_ESM.tif]

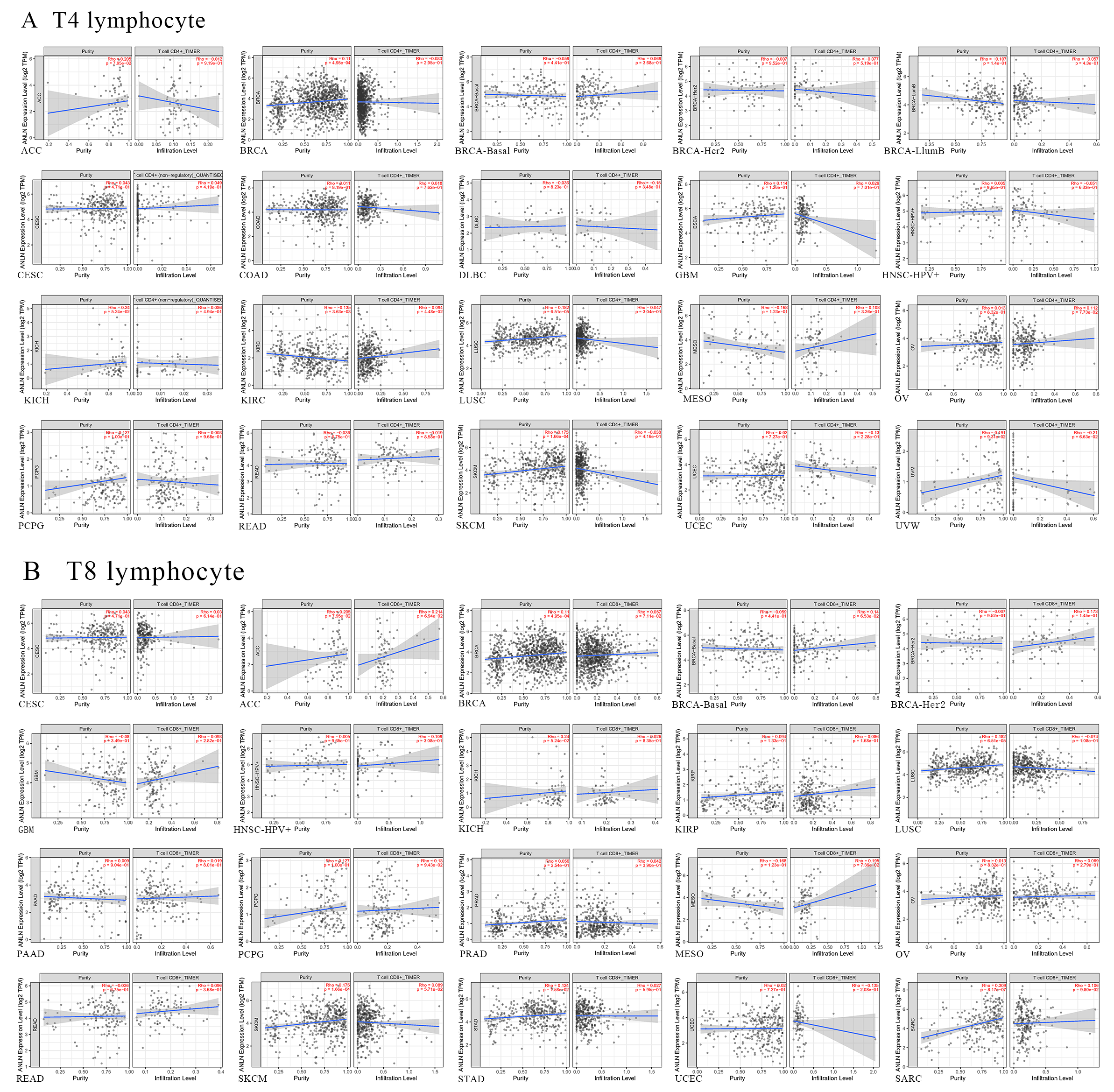

Supplement: Supplementary file 4 — Additional file 4: Fig. S4. Analysis of the correlation between ANLN expression and immune cells. A CD4+T cells. B CD8+T cells. [file 12935_2022_2610_MOESM4_ESM.tif]

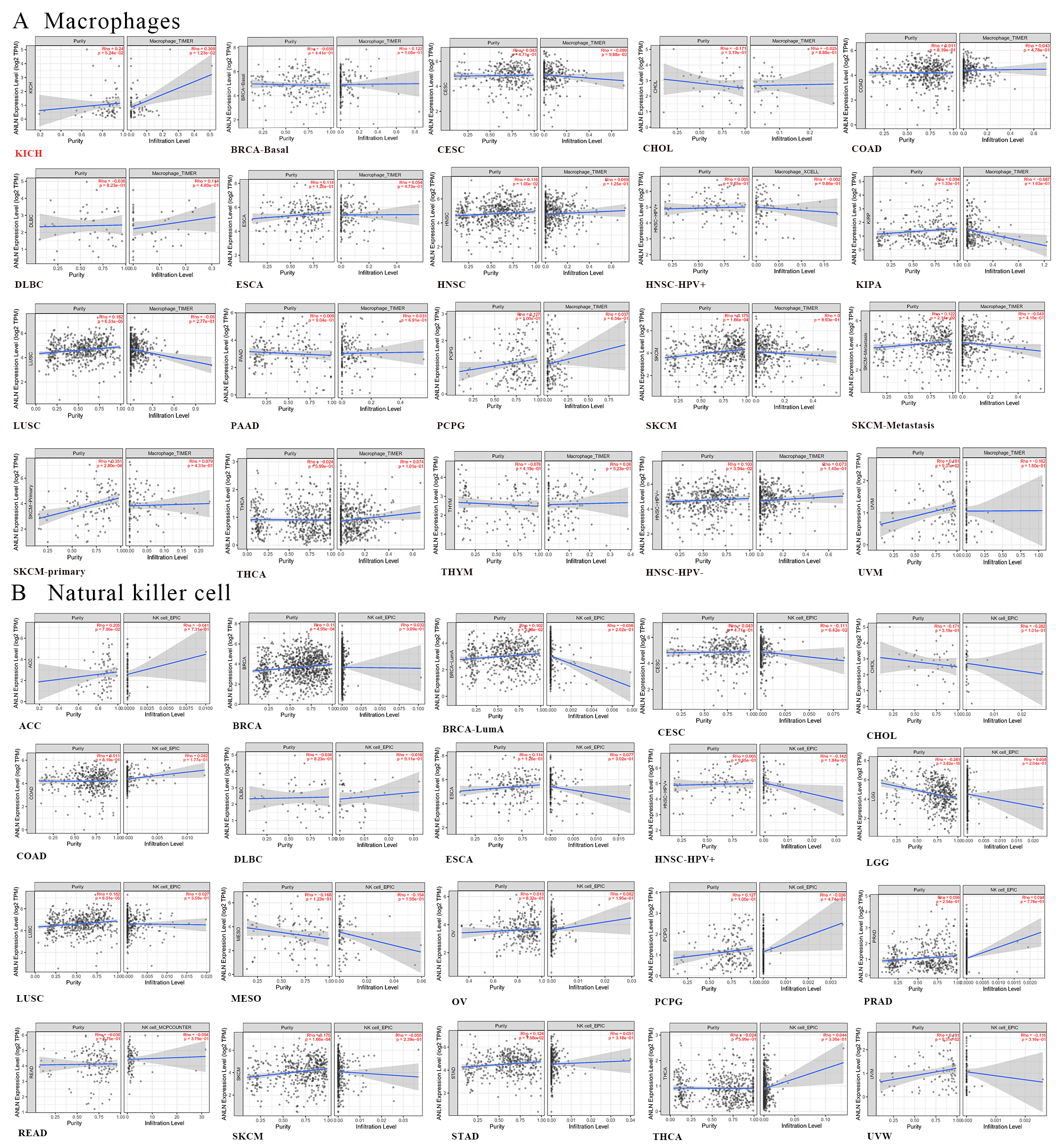

Supplement: Supplementary file 5 — Additional file 5: Fig. S5. Analysis of the correlation between ANLN expression and immune cells. A Macrophage. B NK cells. [file 12935_2022_2610_MOESM5_ESM.tif]

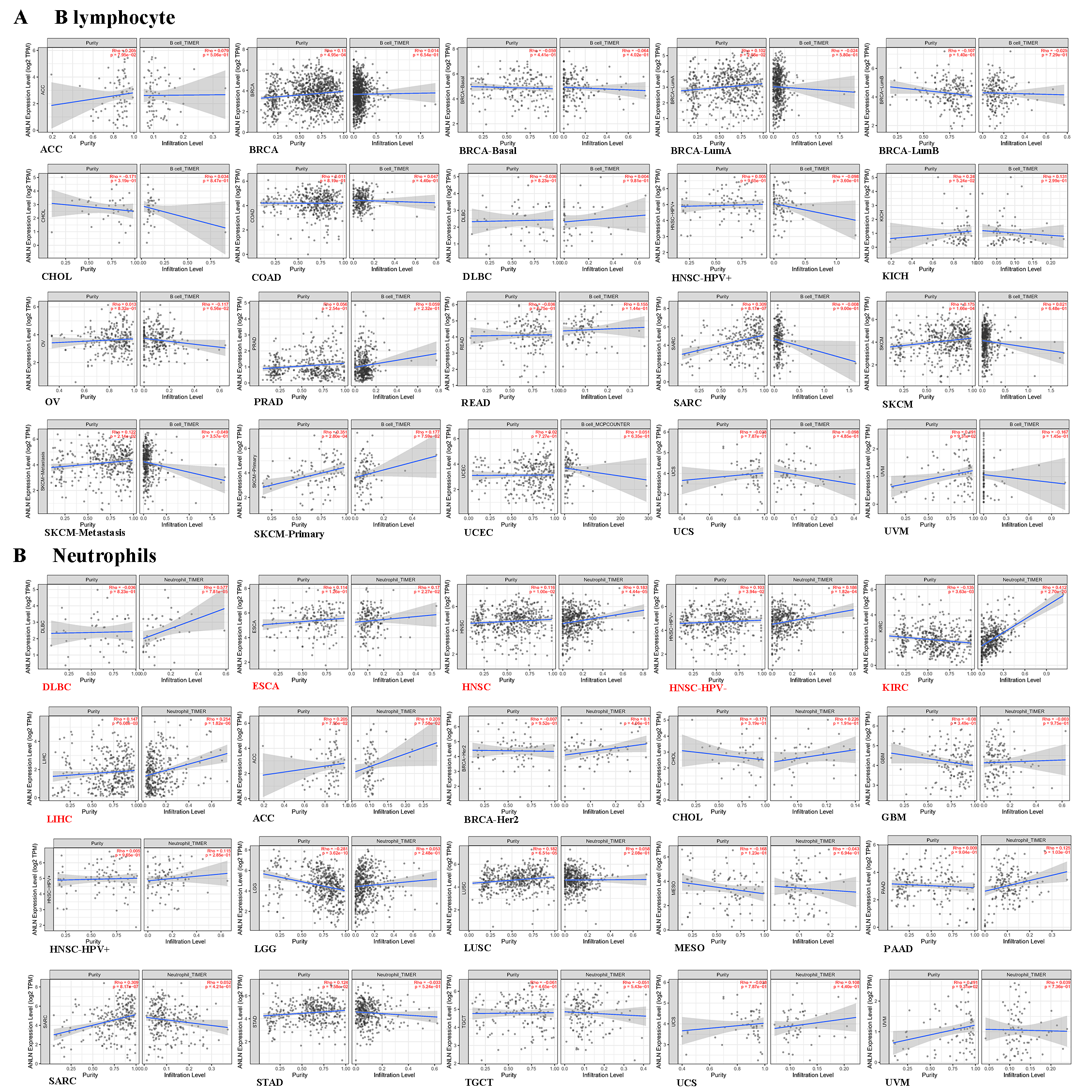

Supplement: Supplementary file 6 — Additional file 6: Fig. S6. Analysis of the correlation between ANLN expression and immune cells. A B cells. B Neutrophils. [file 12935_2022_2610_MOESM6_ESM.tif]
